# Construction of Ecological Security Pattern and Identification of Ecological Restoration Zones in the City of Changchun, China

**DOI:** 10.3390/ijerph20010289

**Published:** 2022-12-24

**Authors:** Jia Xu, Dawei Xu, Chen Qu

**Affiliations:** 1 College of Landscape Architecture, Northeast Forestry University, Harbin 150000, China; 2 Key Lab for Garden Plant Germplasm Development & Landscape Eco-Restoration in Cold Regions of Heilongjiang Province, Harbin 150000, China

**Keywords:** ecological security patterns, circuit theory, ecological restoration zones, Changchun city

## Abstract

Identification of crucial regions in need of ecological conservation and restoration based on ecological security patterns is of utmost importance for ecological restoration across national land space with regard to China’s promotion of ecological civilization. Using Changchun, the capital of northeast China, as an illustration, the study chooses ecological sources based on the importance of ecosystem services, builds an ecological security pattern using circuit theory, and organizes critical regions for ecological conservation and restoration. The findings reveal that the 20 ecological sources chosen based on ecosystem services are more concentrated on the eastern side of the city, whereas the western side of the city has a smaller overall area; 41 ecological corridors show a network distribution, among which the southeast is relatively densely distributed; 31 ecological pinch points and 15 ecological barrier points are also identified. Prioritized restoration zones, prioritized protection zones, key conservation zones, and general conservation zones were the four different types of ecological restoration regions identified by the study. Each district’s prioritized restoration zones in the main metropolitan area are larger than the others; Gongzhuling’s priority protection zones are the largest, and Yushu’s natural substrate is the best. According to the grading, targeted solutions are suggested, offering helpful advice for the improvement of ecological patterns and ecological restoration of the aforementioned national areas.

## 1. Introduction

Cities are now facing major ecological concerns such as resource depletion, declining environmental quality, and declining ecosystem service functions resulting from the accelerated rise in urbanization in recent years. Several ecological and environmental issues, such as biodiversity loss, occurrence of catastrophic climate events, and landscape fragmentation, are becoming increasingly prominent with the rapid development of human civilization [1,2]. The worsening of ecological issues not only has an impact on how sustainably cities develop but also poses a severe threat to human security [3]. As a result, it is critical to maximize the ecological security pattern, designate restoration zones, implement restoration zoning for damaged natural ecosystems, and carry out restoration of crucial habitats and ecologically damaged places.

The ecological security pattern can facilitate coordination between the expansion of ecosystem services and socioeconomic development [4], increase the capacity of ecosystem services, encourage rational resource allocation, and ensure the security of urban ecosystems by adjusting and improving the spatial organization of cities and restoring ecologically vulnerable areas [5]. The rational building of an ecological security pattern can bolster the protection of environmentally fragile places, actualize the sustainable use of regional natural resources, and foster high-quality urban growth. Initially, the construction of ecological security patterns was based on qualitative planning and quantitative pattern analysis of space, primarily for biodiversity protection [6]. However, as ecosystem service evaluation developed and people’s understanding of ecological security pattern grew, the focus of research has shifted towards ecological processes and functions. These include spatial evolution research that spatially identifies and annually analyzes ecological security patterns [7], optimization of static patterns research that identifies and proposes optimization measures for ecological security patterns [8], simulation of dynamic patterns research that extracts annual ecological security patterns and simulates future patterns based on optimization suggestions [9], and analysis of state trends research that evaluates the year-by-year state of ecological security patterns and analyzes future trends [10]. The identification and restoration of ecologically fragile areas, as well as the formulation of regional ecological security policies based on the construction of ecological security patterns, serve as a guide for restoring ecological service functions, bolstering the resilience of ecological structures, and achieving the goal of restoring the regional ecological environment.

The study of ecological security patterns has gradually led to the formation of a fundamental paradigm characterized by “ecological source identification, resistant surface construction, and corridor extraction [11]”. This prevalent paradigm has been widely implemented in cities [12], mineral resource areas [13], drainage basins [14], and arid zones [15], among other places, and it can precisely regulate and maintain regional ecological security. Ecological sources are significant ecological patches with radiative functions, and one method of identifying ecological sources is based on the qualitative evaluation of regional ecosystem structures [16], selecting areas with high ecological benefits such as nature reserves, forests, and wetlands while ignoring dynamic changes in intrinsic ecological quality. The alternative method is a thorough quantitative evaluation based on ecological connectivity, ecological sensitivity, and ecological relevance indicators, which is more in accordance with the ecological substrate and development status of the research area. Among them, the ecosystem services importance assessment can express the provisioning, regulating, supporting, and cultural services that ecosystems provide to humans, integrating the ecological significance of complex ecological issues and indicators, and is used in numerous studies [17,18,19] to select ecological sources. The resistance surface can express the difficulty of species migration and energy flow across heterogeneous landscapes, and is constructed according to species migration patterns and analyzed by diffusion models [20], typically based on the use of principal component analysis to calculate the weights according to the characteristics of the study area to select the land type, topography, urban POI points, and other indicator factors overlay analysis [21,22]. Some studies have employed nocturnal illumination intensity (NLI) to rectify resistance face values and to thoroughly quantify the effect of anthropogenic influences on species migration [23]. Ecological corridors are essential biological components that connect the region’s ecological processes and activities. These present the pathway of biological circulation and exchange between ecological sources, and the minimum cumulative resistance (MCR) model and circuit theory [24] are primarily used for ecological corridor extraction. The corridor retrieved by the least cumulative resistance model may determine corridor importance using the gravity model matrix; however, corridor width cannot be directly estimated. By modeling charge transportation, the circuit theory model can accurately calculate corridor width, determine corridor importance, and designate the ecological protection zones by ecological key nodes [25]. Ecological key nodes consist of ecological pinch points and ecological barrier points, in which ecological pinch points are nodes of high ecological flow located in the corridor that play an important role, and ecological barrier points are regions with a high level of resistance to biological circulation and exchange flows, and are frequently regarded as conflict zones in contemporary land development and ecological protection [26]. Source centrality, which indicates the significance of ecological sources in influencing their neighboring patches and connected corridors, has been utilized more frequently in recent studies to determine the significance of ecological sources in the construction of ecological security patterns [27].

Presently, ecological restoration continues to attract increasing attention, and the key to achieving spatial ecological restoration is to scientifically identify the ecological restoration zones to be restored, propose ecological restoration strategies, and implement conservation and restoration projects in ecological restoration zones in a hierarchical fashion. The identification of territorial spatial restoration regions has been a prominent topic of research [28] in China aimed at the development of territorial spatial planning. The identification of major ecological pinch points and ecological barrier points based on circuit theory can provide a scientific basis for urban territorial spatial planning through the construction of ecological protection patterns and the identification of major restoration areas from a global perspective within the city [16]. China’s 14th Five-Year Plan calls for the driving role of central cities and urban agglomerations. Changchun, as the central city of the Northeast Asian Economic Circle, is facing a conflict between urban development and ecological protection as the demands of rapid urbanization continue to grow [29]. In order to implement the important development strategy regarding the comprehensive revitalization of northeastern China, Gongzhuling City was divided into the escrow area of Changchun City in 2020, and Changchun City both faced with severe challenges and valuable development opportunities in the new round of spatial planning. This paper uses Changchun as the study area, quantifies the significance of four ecosystem service functions through ecosystem service evaluation, identifies ecological sources that guarantee regional ecological security; constructs a resistance surface based on regional characteristics, corrects the resistance surface calculation results using nighttime lighting intensity data, calculates ecological corridors, and constructs an ecological corridor network. On this basis, ecological pinch points and ecological barrier points are identified, and ecological restoration regions are subdivided for the protection and restoration of vital ecosystems. For the future trade-off between land management and ecological conservation, optimization recommendations for graded restoration zones are also provided. Suggestions are presented for enhancing the management of ecological restoration in urban settings.

## 2. Materials and Methods

### 2.1. Study Area

**Figure 1:**
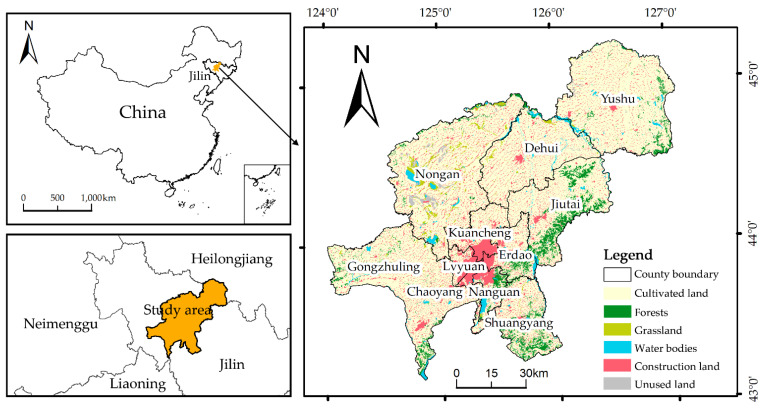
Location and land use types of the study area.

Changchun City is located in central Jilin Province, (124°18′–127°05′ E, 43°05′–45°15′ N), Changchun under the jurisdiction of 7 districts (Nanguan District, Kuancheng District, Chaoyang District, Erdao District, Luyuan District, Shuangyang District, Jutai District), one county (Nongan County), and three county-level cities (Yushu City, Dehui City, and Gongzhuling City) under its jurisdiction, with a total area of 24,744 square kilometers. Changchun has several rivers and lakes, significant water resources, limited forest resources, and a smaller forest land area than the province and national averages. The figure depicts Changchun City’s study area and land use types (Figure 1). Changchun is an important hub of industrial development and an important grain production base in northeast China, a typical location of fast urbanization and resource and environmental changes in northeast China, and a key region for “northeast China revitalization” and “The Belt and Road Initiative.” The construction of the China–Mongolia–Russia Economic Corridor and the development of related industries have fueled the city’s economy, and rapid economic development has led to the expansion of urban space and construction land, which urgently require resource integration and optimization of ecological security pattern, as well as the identification and protection of ecologically significant areas, to coordinate urban economic development and ecological security.

### 2.2. Data Sources and Pre-Processing

The land use data of Changchun City in 2020 for this study were obtained from the Resource and Environmental Science and Data Center of the Chinese Academy of Sciences (https://www.resdc.cn, (accessed on 10 March 2022)); the DEM 30 m resolution digital elevation data and Landsat 8 OLI data were obtained from the Geospatial Data Cloud (http://www.gscloud.cn, (accessed on 4 November 2021)), the DEM data were processed to extract the slope and elevation of the study area. In addition, Landsat 8 OLI data were pre-processed with geometric correction, radiometric calibration, and atmospheric correction, then normalized difference vegetation index (NDVI) data were generated; the soil data were obtained from the National Center for Glacial Tundra Science and Desert Data World Soil Dataset (http://www.ncdc.ac.cn, (accessed on 7 March 2022)); the meteorological data were obtained from China Meteorological Data Network (http://data.cma.cn (accessed on 10 March 2022)), which contains annual rainfall and temperature data, and based on the rainfall and temperature data from meteorological stations in the study area, rainfall and evapotranspiration data were processed by spatial interpolation. The nighttime lighting intensity data were obtained from the official website of the National Oceanic and Atmospheric Administration (https://www.noaa.gov, (accessed on 24 March 2022)), and the monthly average light radiation data in 2020 were selected and processed into annual average data; The Resource and Environment Science Data Center of the Chinese Academy of Sciences provided us with traffic data and administrative boundaries. (http://www.resdc.cn, (accessed on 10 March 2022)). In this study, the raster data resolution was processed into 30 m and all coordinate systems were unified to ensure spatial reference consistency.

### 2.3. Methods

#### 2.3.1. Evaluation of the Importance of Ecosystem Service Functions 

Ecosystem services can express the multifunctionality of ecosystems and maximize the benefits of protecting both people and nature [18]. The Northeast is rich in wildlife resources and provides a habitat and migration point for many rare animals, and it is important to provide living space for wildlife. Some areas of Changchun are relatively saline and sandy soils, creating serious water loss and soil erosion problems. At the same time, carbon storage should also be listed as an important ecosystem service function to achieve the emission peak and carbon neutrality targets set by the Chinese government. Considering the above conditions combined with the current ecological environment of the Changchun city area, the ecosystem services of the Changchun city area were evaluated in terms of four dominant service functions: biodiversity, water supply, soil conservation, and carbon storage. The InVEST model based on the formation mechanism and action process of ecosystem services was used for the evaluation.

1. Functional evaluation of biodiversity

Based on the habitat quality model in the InVEST model to calculate the habitat quality index to express biodiversity in Changchun [30], the final selection of paddy fields, drylands, urban land, rural settlements, and another construction land as threat sources was made by referring to the instructions for using the model and related studies [31,32], and the relative sensitivity of the LULC secondary classification to threat sources was determined according to the actual situation of the area. The calculation formula is as follows.

(1) $$Q_{xj}=H_{j}\left[1-\left(\frac{D_{xj}^{z}}{D_{xj+k^{z}}^{z}}\right)\right]$$

where *Q_xj_* denotes the habitat quality in *j* habitat types corresponding to raster *x*; *H_j_* is the habitat suitability of habitat type *j*; *D_xj_* is the habitat degradation of raster *x*; *z* is the scale constant, and *k* is the half-saturation constant.

2. Evaluation of water supply function

The water supply service is evaluated based on the InVEST model. The model calculates water supply based on the Budyko hydrothermal coupling equilibrium assumption [33], which is calculated as follows.

(2) $$Y_{xj}=\left(1-\frac{AET_{xj}}{P_{x}}\right)\times P_{x}$$

(3) $$\frac{AET_{xj}}{c}=1+\frac{PET_{xj}}{P_{x}}-{\left[1+{\left(\frac{PET_{xj}}{P_{x}}\right)}^{\omega }\right]}^{1/\omega }$$

where *Y_xj_* represents the annual water production; *AET_xj_* is the actual average annual evapotranspiration of raster x on land use type *j*; *P_x_* is the average annual rainfall of raster *x*; *PET_xj_* is the potential evapotranspiration of raster *i*; *w* represents the available water content of vegetation.

3. Evaluation of soil conservation function

The soil conservation model based on the InVEST model represents the role of the ecosystem in reducing soil erosion due to water erosion through its structure and processes by the difference between potential and actual erosion [34,35]. The calculation equation is as follows.

(4) $$SD_{i}=RKLS_{i}-USLE_{i}$$

(5) $$RKLS_{i}=R_{i}\times k_{i}\times LS_{i}$$

(6) $$USLE_{i}=R_{i}\times k_{i}\times Ls_{i}\times C_{i}\times P_{i}$$

where *SD_i_* denotes soil conservation amount; *RKLS_i_* is potential erosion amount; *USLE_i_* is actual erosion amount; *R_i_* is rainfall erosion force factor; $k_{i}$ is soil erodibility factor; *Ls_i_* is slope length factor; *C_i_* is management factor for plant cover; *P_i_* is soil and water conservation measure factor.

4. Functional evaluation of carbon stock

Based on the soil conservation model of InVEST, a carbon pool was established by combining land use data with the real-life situation of the study area and references from related studies [36] to calculate the regional carbon content. The calculation equation is as follows.

(7) $$C_{total}=C_{soil}+C_{above}+C_{below}+C_{dead}$$

where *C_total_* is the regional carbon stock; *C_soil_* is the soil carbon stock; *C_above_* is the above-ground carbon stock; *C_below_* is the below-ground carbon stock; *C_dead_* is the dead organic matter carbon stock.

#### 2.3.2. The Identification of Ecological Sources 

Ecological sources can provide ecosystem services and prevent ecosystem deterioration owing to their radiative functions [37]. Important areas of integrated ecosystem service evaluation were employed as ecological sources in this study, and the results of each function of ecosystem services were standardized by extreme difference to remove the influence of the base. Due to the interaction between the ecosystem service functions, it is possible to account for the mutual influence of each function and establish the weights among the ecosystem service functions more precisely using the main component factor analysis method [38,39]. The coefficients of various functions in each linear combination rate of principal components were weighted and averaged to obtain the contribution of the variance of the principal components, and the weights of various ecosystem service functions were obtained by extracting spatial point data to calculate multivariate principal component factors [40]. The results of the biodiversity, water supply, soil conservation, and carbon storage indicators were divided into 5 km × 5 km fishing nets using ArcGIS10.8 after the standardization of extreme differences. The center point value of each fishing net was determined, and the boundary value was eliminated. Utilizing SPSS27.0’s principal component factor analysis software, 987 data sets were computed and weights were determined. The measured KMO value was 0.62, suggesting that the correlation between ecosystem services was robust and satisfied the requirement for main component factor analysis. The weights were determined to be 0.28, 0.22, 0.20, and 0.30, respectively, and the overlaid analysis classified them as extremely significant, very important, important, moderately important, and generally important using the natural breakpoint approach. Taking into account the regional characteristics of the study area, patches with highly important alternative sites for ecosystem services larger than 10 km^2^ were chosen as ecological sources [41].

#### 2.3.3. The Construction of Basic Resistance Surface

The ecological resistance surface reflects the trend and possibilities of landscape ecological functions and ecological spatial processes by expressing the different resistance factors of species migration and energy flow [17]. According to pertinent research [23,42] and the features of the ecological land distribution in Changchun, the resistance factors for constructing the resistance surface included land use type, elevation, slope, and normalized vegetation different index (NDVI). Of these, land use type and elevation can control the soil and hydrological conditions that determine the spatial landscape differentiation of the area, the slope may affect the ease of animal migration and energy flow, and the crop growth and nutrient information expressed by NDVI can determine the migration path of species. Referring to relevant research [43], a hierarchical analysis was used to establish a discriminant matrix of resistance components to determine the weights of the four resistance factors. While the resistance variables were further valued with reference to pertinent studies in northeastern China [44,45,46], taking into account the habitat characteristics of species in cold parts of China and the variations in ecological suitability of the study area (Table 1). Then use ArcGIS10.8 to superimpose the weights obtained on each resistance surface to get a comprehensive resistance surface. The comprehensive resistance surface accounted for objective factors of the area but did not account for the influence of human activities on species exchange and energy flow [47], so this study used nighttime lighting intensity data, which characterize the degree of human activity interference, to correct the ecological resistance factor in order to improve the accuracy and rationality of corridor extraction.

#### 2.3.4. Constructing Ecological Restoration Zones 

In this study, we employ circuit theory to extract ecological corridors and identify ecological pinch points and barrier points, and then calculate the centrality of ecological source points to form ecological safety patterns and demarcate ecological restoration regions in Changchun. Circuit theory is used to locate the lowest resistance corridors between sources and locations by modeling electron random walks to determine ecological corridors. Linkage Mapper [48] was used to extract ecological corridors based on a threshold setting of ecological corridor protection area within 20% of the study region, while the critical corridors are designated as general conservation zones.

Landscape connection can be addressed to the maximum extent by recovering the barrier regions through ecological restoration. Ecological pinch points are identified using the Pinchpoint Mapper tool, and for maintaining the stability of corridor circulation, ecological pinch points are designated as Prioritized protection zones. Ecological barrier points are identified using the Barrier Mapper tool, and for maintaining the corridor’s connectivity, ecological barrier points are designated as Prioritized restoration zones. The importance degree of one ecological source in an ecological security pattern can be described by the source centrality, while the Centrality Mapper program was used to repeatedly calculate the cumulative electric current using the current ecological source as the node, and the ecological source with the top fifty percent of the cumulative current value was chosen as the key conservation zones [49]. Based on the aforementioned techniques, Changchun’s ecological restoration zones are classified hierarchically according to the level of protection and restoration zones’ necessity.

## 3. Results

### 3.1. Source Identification Based on the Importance of Ecosystem Services

**Figure 2:**
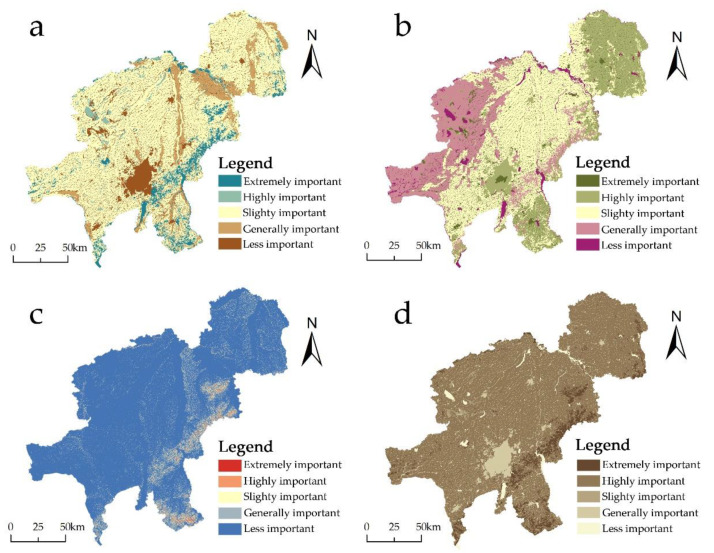
Four types of ecosystem service importance levels. (**a**) Biodiversity importance rating. (**b**) Water production importance rating. (**c**) Soil conservation importance rating. (**d**) Carbon fixation importance rating.

In Changchun, the spatial distributions of biodiversity, water production, soil conservation, and carbon fixation were determined using the InVEST model to account for the four categories of ecosystem service activities. The results indicated that the four ecosystem service functions exhibited spatial heterogeneity, and each function was subdivided into five classes for analysis (Table 2), including: the spatial heterogeneity of biodiversity is evident, and the extremely important areas are concentrated in the south side of the city with a proportion of 7.24%. They contain a dense vegetative cover, a diversity of species, minimal human involvement, and high ecological environment quality. In the water production service, the places with higher topographic relief are generally higher than the plains and flat terrain areas, and the western and eastern parts are also higher than the central regions (Figure 2a). The bulk of extremely important locations are situated on the head slopes of the Great Black Mountains, where the terrain is extensive and precipitation is quite high, resulting in an overall high water production capacity. In the middle region, both highly and moderately significant regions are dispersed. Among these, the spatial extent of the generally important areas is the greatest at 44.53%, showing that this city is a water scarce region (Figure 2b). Soil conservation extremely important are in Changchun is mostly concentrated on the city’s southeast side, which accounts for 1.15% of the overall study area, and steadily decreases in the surrounding areas. Except for the extremely important areas, the slightly important and highly important areas made up 18.83% of the entire area, showing that the general soil conservation capacity of the total area is quite low. The study area comprises a large arable land area, copious precipitation during the rainy season, and significant soil erosive causes, as well as insufficient precipitation regulation and storage capacity in most locations; thus, the study area has poor water conservation and soil conservation capacity (Figure 2c). The carbon fixation service is mostly located on the south side of the city, with the extremely important areas comprising 6.73% of the total area. The majority of land use categories in these regions are wooded areas with abundant vegetation cover, humid climates, and favorable ecological circumstances, which are more able to support plant growth than other habitats. Highly and slightly important areas are more widely dispersed, encompassing the majority of the municipal territory, and consist primarily of arable land, water bodies, and other land use categories. The less important areas contain the greatest construction land and rural settlements (Figure 2d).

**Figure 3:**
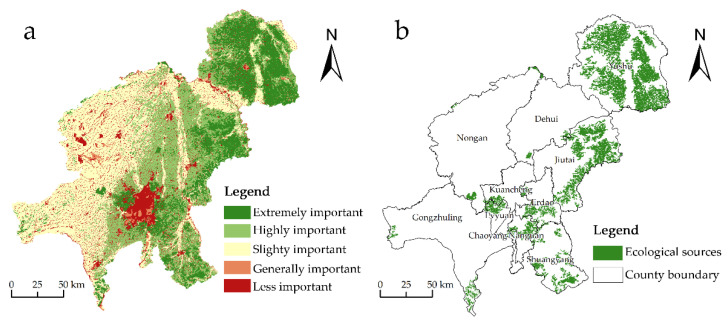
Ecosystem service importance and ecological sources screening. (**a**) Ecosystem service importance rating. (**b**) Ecosystem sources.

The evaluation results of the four functions of biodiversity, water production, soil conservation, and carbon fixation were superimposed on the weights calculated via principal component analysis to obtain evaluation results of the importance of ecosystem services into five levels (Figure 3a), in which the area of the extremely important area of ecosystem services is 4698.92 km^2^, accounting for 18.99%; the area of the highly important area of the ecosystem is 6941 km^2^, accounting for 28.05%;.The extremely important and highly important ecosystem service areas are primarily cultivated land and forests, which are mainly located in the east of the city and have significant water supply and biodiversity functions to provide a strong guarantee for urban nutrient maintenance. The slightly important ecosystem covers 9363.97 km^2^, or 39.46% of the total area, and consists primarily of cultivated land that is widely dispersed throughout all of the city’s counties and districts. This ecosystem provides essential ecological services for the city’s healthy and stable development. The total area of generally important and less important ecosystem service areas is 3340.80 km^2^, or 13.50%, and is primarily distributed in urban construction areas and sporadic walks along the periphery of construction sites whose ecosystem services must be enhanced. Using ecological service evaluation and area rationalization configuration, the study identifies the 20 ecological sources with a total area of 3659.28 km^2^, accounting for 14.79% of ecological land, and identifying the patches of alternative land larger than 6 km^2^ that provide these ecological sources. The ecological source land types consist primarily of agricultural land, followed by forests and grassland, with almost no ecological source for construction land, indicating that human interference has a significant impact on the natural ecosystem (Figure 3b). This is due to the fact that the Changchun city limits contain a large area of arable land and a more concentrated area of forest land. According to the distribution of ecological sources, Changchun’s ecological sources are distributed in the eastern plain and southern Daheishan Mountain Ridge regions, with larger areas in Yushu City, Jiutai District, Shuangyang District, and Erdao District. These regions have superior natural resource conditions and are distinguished by fertile soil and less human interference.

### 3.2. Construction of Resistance Surface

**Figure 4:**
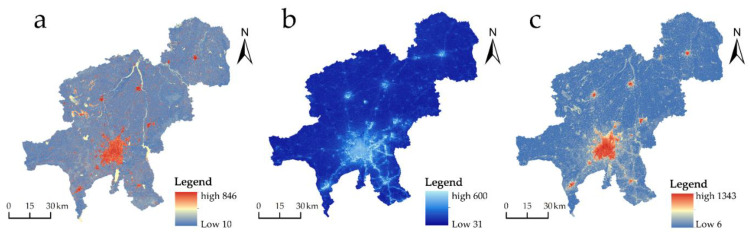
Ecological resistance surface. (**a**) Spatial distribution of the basic resistance surface. (**b**) The spatial distribution of nighttime light intensity (TLI). (**c**) The spatial distribution of the modified resistance surface.

The fundamental resistance surface is derived from the superposition of resistance factors and weights, whereas the ecological resistance surface is derived from the correction of nighttime lighting intensity (TLI) (Figure 4). The surface of corrected resistance expresses the spatial distribution of resistance that is composed of both natural resistance factors and resistance factors. The modest cumulative resistance values indicate the spatial resistance values as well as show the selectivity of ecological sources on habitats and the level of landscape disturbance to species. In Changchun, the spatial distribution of the integrated resistance surface is highly heterogeneous, with the resistance being greater in urban centers compared to the entire spatial distribution. The tendency of high resistance values have shifted from the county’s core to its outskirts. Due to the limited availability of marshland and beach land types, the high-resistance zones in the southern region are unevenly distributed. In addition, the higher elevation of mountain ranges in the southern Jutai region bear a steeper slope due to their position and topography, resulting in an overall trend of higher relative resistance values than the surrounding areas.

### 3.3. Corridor Extraction and Ecological Node Identification

Using Linkage Mapper to extract the paths with the lowest cost to establish ecological corridors, a total of 41 important ecological corridors and 7 potential ecological corridors were created in 20 ecological sources in Changchun. The entire length of important biological corridors measures 1397.90 km, with the shortest measuring 0.40 km and the longest 141.85 km. The distribution of critical ecological corridors in Changchun city forms a more uniform network, and the distribution of key corridors is relatively concentrated in the city’s southern region. The ratio of cost-weighted distance (CWD) to the least-cost path to its length (LCPL) can be used to quantify the corridor cost, and the bigger the ratio, the more resistant ecological corridors are to species migration between ecological source locations. The average CWD: LCDL value of 70.47 for the important ecological corridors suggests strong overall connectedness; however, the lowest ratio of 47.60 for the easternmost ecological corridor in Erdao district and Shuangyang district necessitates ecological protection of the corridor.

**Figure 5:**
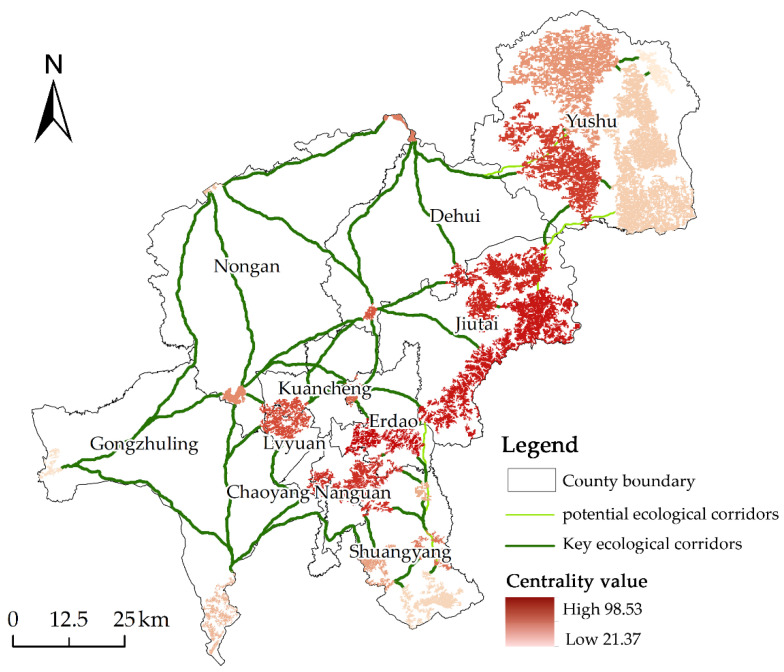
Ecological Source Centrality and Ecological Corridors.

Centrality Mapper was able to determine the ranking and distribution of ecological sources based on its analysis (Figure 5). The darker hue of the ecological sources denotes a higher cumulative current value and a more powerful center. The ecological source in Erdao District has the highest cumulative current value of 98.53, which is the core area of ecological sources and contributes the most value to regional ecological source connectivity. Following this are the two natural sources located in the Jiutai District. The ecological sources with weaker centrality are situated in the eastern portion of Yushu City and the western portion of Gongzhuling City, with cumulative current values of 21.37 and 26.49, respectively, which have a lesser effect on the overall connectedness.

**Figure 6:**
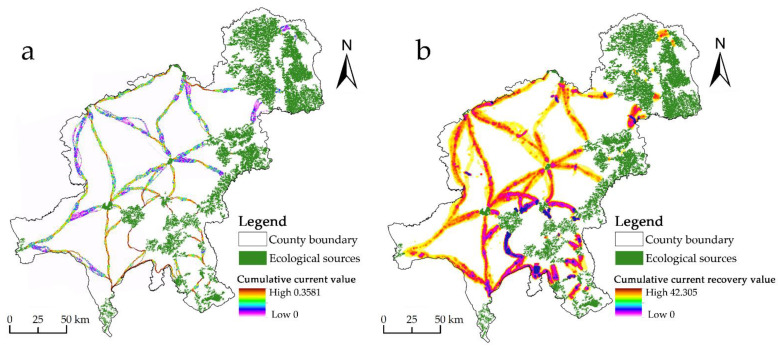
(**a**) Distribution of ecological pinch points. (**b**) Distribution of ecological barrier points.

The current intensity distribution of Changchun’s ecological corridors was derived using the ecological security pattern (Figure 6a). In total, 35 ecological pinch points covering 109.08 km^2^ were found. The ecological pinch points were striped, and 81.28% of the research region consisted primarily of agricultural land. It is related to a high-centered ecological source in Chaoyang District, which is an essential region for communicating with the source patches and has high ecological value. Based on the corridor construction, 15 ecological barriers with a total size of 131.6 km^2^ were discovered (Figure 6b). With an area of 23.34 km^2^, the greatest barrier point is located on the west side of the green park. A 34.41 km^2^ area of the barrier points are located on the ecological corridor, which has a significant impact on the regional landscape connectivity because the land type of these barrier points consists primarily of cultivated land and construction land, accounting for 48.62% and 36.3% of the study area, respectively, leading to greater resistance to the extension of the ecological corridor in this area. The biological substrate of the corridor is difficult to maintain, hence creating a barrier point area in the research region. 

### 3.4. The Construction of Ecological Restoration Zones

**Figure 7:**
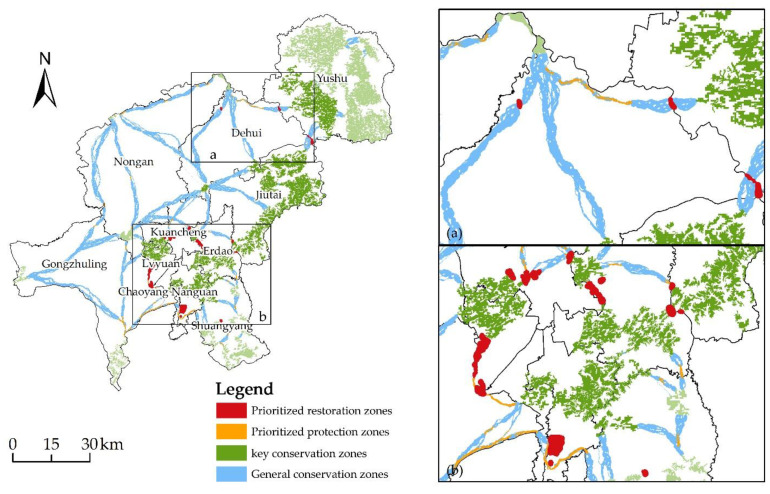
Ecological restoration zones ((**a**,**b**), are partially enlarged views).

The ecological functional zoning covering the functional basis zoning of the study area served as the basis for identifying ecologically graded protection and restoration areas in Changchun, taking into account ecological substrate, natural resources, and the distribution of ecological security pattern components. The ecological pattern elements were ranked based on priority restoration and hierarchical protection, as prioritized restoration zones, prioritized protection zones, key conservation zones, and general conservation zones (Figure 7), and the restoration areas of different districts and counties were counted in detail (Table 3); the identification of restoration areas in different districts and counties can play a more accurate role in the ecological optimization of the overall ecological security pattern, and the ecological restoration zones of Changchun city were determined based on the ecological connectivity and stability of the entire area. 

The prioritized restoration zones are mainly concentrated in the main urban part of the city, which includes Kuancheng District, Erdao District, Lvyuan District, Chaoyang District, and Nanguan District, and another small area is gathered in Dehui City, Nongan County, and Gongzhuling City, mainly because the total population of the town is concentrated in the main urban area and human interference and activities have a greater impact on the overall connectivity of the corridor in the main urban area.

The prioritized protection zones are distributed relatively evenly throughout each region, with the largest area located in Gongzhuling City. Priority protection zones must be established in each of the three ecological corridors connecting the ecological source points in the southern region of Gongzhuling City. Within the urban region of Yushu City, natural circumstances are favorable and ecological sources are widely dispersed. Due to its location downstream of the Liaohe River basin, where soil and water conservation are of utmost importance, this land is not a priority protection zone.

The key conservation zones are ecological source points with significant cumulative currents and strong ecological radiative advantages to the surrounding area. The largest area of distribution in Jiutai District is due to the rich biodiversity of the Daheishan Mountain Ridge area distributed in Jiutai District, less disturbance caused by human activities, and the notable width of its surrounding corridor. Yushu City has a greater number of major conservation zones, primarily grassland, and cultivated land. The critical protection areas in Erdao District and Kuancheng District within the main urban area are larger, consisting primarily of the transition zone between natural and human populations in the urban fringe area, which have a fragile ecological environment and are easily degraded due to pressure exerted by human activities; thus, they require targeted protection.

The general conservation zones are ecological corridors identified in Changchun city, which constitute the basic network architecture of ecological security patterns but are also subject to intense pressure from declining ecosystem functions and human activity. Due to the absence of hubs and the high construction cost distance of the corridors, Nongan County has the biggest protected area, making biological dissemination and migration more susceptible to threats. Due to the influence of water systems and topographic changes, Yushu City and Jiutai District have a wide amount of protected areas with a variety of plant species and land use types, and the ecological corridor is relatively distant. 

## 4. Discussion

### 4.1. Spatial Heterogeneity of Ecosystem Services

Ecosystem service functions are closely related to ecosystem changes, and the construction of ecological security patterns relies on the identification and control of ecosystem services. As shown in Figure 3, each key ecosystem service and integrated ecosystem in Changchun exhibits obvious spatial heterogeneity due to the influence of the natural environment and human activities. Analysis based on land use types: land use types in Changchun show that cultivated land occupies the most area throughout the city, which stems from the fact that Changchun is an important city for grain production. The overall ecosystem services in the eastern part of the city are higher than those in the western part due to the fact that the eastern part is located on the western side of the Changbai Mountain Range, which is relatively rich in forest land resources and has radiative benefits for biodiversity, soil and water conservation, and carbon fixation within the city, as these regions are essential for the migration routes and habitats of nationally protected plants and animals. On the other hand, Nongan County located on the west side is in the less important area for all four services due to salinization of the agricultural land. The spatial differentiation of ecosystem services is also influenced a little by geographical factors; Changchun is in the Songliao Plain area [50], and the east side is influenced by the Daheishan Mountain Ridge and Jilin Hadar Mountain Ridge with overall high elevation, while the west side of Nongan County and Dehui City is located in the Songhua River Basin with weaker relative water supply and soil conservation functions, resulting in the weaker importance of ecosystem services in the west side. The greatest impact on ecosystem services comes from anthropogenic [51], where the less important areas are distributed in urban centers where humans gather activities, where construction sites are affected by human activities and impermeable surfaces reduce water infiltration and storage, thus negatively affecting soil and water conservation, and blocking animal migration and plant distribution, of all which bear huge impacts on biodiversity [52]. Ecologically less important areas are located on constructed land and affect the surrounding area, requiring strict control of urban sprawl and changes in the nature of ecological land.

### 4.2. Ecological Protection and Restoration

Changchun, as the provincial capital of Jilin, is a crucial area for the implementation of China’s National New Urbanization Plan (2021–2035). Its development stimulates the revival plan of the historic industrial bases in Northeast China, the integration of regional economic development, and urban transformation. Despite Changchun’s fast economic transformation, the ecological environment remains under increasing pressure. The linkage between ecological sources has also been hampered by large urban built-up regions and fragmented, intermittent concentrations of human life brought on by urban development and frequent human activities [53]. Therefore, identifying the ecological security pattern and ecological restoration for the city is essential. In response to the challenges in Changchun City, it is required to design an ecological security pattern to restore the ecological space of the national land area. Since Gongzhuling City joined the Changchun planning area as the Changchun neighborhood in 2020, Changchun requires ecological restoration strategies that meet the requirements and positioning of urban development and provide a basis for the overall land space planning of the new planning area. The government work report includes additional requirements for scientifically identifying ecological protection red lines and speeding the creation of a new development and preservation pattern for Changchun’s national land space. Based on Changchun’s ecological connection and stability, multiple restoration solutions were presented for four ecological restoration zones.

For Prioritized restoration, zones rehabilitation can be conducted in accordance with the city function zone [54]. For the construction land within cities, urban green areas such as green belts and green centers can be strengthened to increase the abundance of vegetation and ensure continuity of the ecosystem within the city [55]; for cultivated land areas, green space planning and appropriate reforestation can be implemented. There is a need to avoid and control the salinization of arable land in Nongan County and Dehui City, actively manage and firmly control it, and adhere to the principle of agricultural land use regulation. Strengthen the ecological stability of the relationship between source land and corridor.

For Prioritized protection zones, as a topographic depression area of Songliao, Dehui City must prioritize protecting the ecological corridor on the north side. Furthermore, as an important corridor connecting the ecological sources of Yushu City and Nongan County, it must plan the regional green areas, optimize urban and rural green areas, and expand green space.

For Key conservation zones, appropriate protective measures must be developed for priority protection, by enhancing the ecological circulation as a whole. Jiutai District is the largest area of ecological sources distribution that needs to consolidate the existing ecological foundation, focus on ecological engineering led by woodland biodiversity conservation, and pay close attention to the dynamic balance of forest growth and harvesting utilization [56]. The key conservation zones of Yushu City must intensify vegetation cultivation to provide adequate ecological space for the migration, habitat, and reproduction of species.

For General conservation zones, these serve as a conduit for resource and energy transfer [26]. Attempts can be made to preserve the biological land in this region in order to increase the possibility of inter-source communication and allow corridor expansion [57]. To ensure the connectivity of biological corridors, it is necessary to pay more attention to Nongan County which has a larger area of ecological corridor and increase their preservation. In addition, regarding the districts in the main urban area, consideration should be given to the occupation of ecological corridors during urban development. This requires the construction of green areas around construction sites and timely restoration of damaged land in order to specifically eliminate the negative factors of connectivity, thereby enhancing the status of ecological corridors and their ecosystem services.

The restoration areas identified in this study were assessed in terms of Changchun’s terrain, vegetation, and human influence. Changchun’s topography is typically flat, with low hills in the east and river floodplains in the northeast. Favorable topographic conditions can minimize ecological restoration difficulties in the area. The greatest forms of vegetation in Changchun include forests, scrub, grasslands, and planted green areas: forests, including natural forests and natural forests, are situated primarily in the eastern low hills, the study did not discover any forest that need to be restored, but they should be conserved. Thickets are primarily distributed along the riverbanks of Jiutai District and Yushu City; their overall ecological conditions are favorable, and protection in conjunction with the restoration areas identified in the study can effectively improve ecological benefits; grassland vegetation is primarily distributed in the southern portion of Nongan County; restoration of grassland vegetation in conjunction with the identified restoration zones can improve the overall ecosystem. Priority restoration locations are primarily located in urban cores, which must be restored in a timely manner. Changchun’s ecological security pattern is more influenced by human activities, and the Prioritized restoration zones identified in the study are primarily concentrated in the centers of cities and counties; the construction of roads has obstructed the original ecological corridors, necessitating the re-planning of urban green space.

Combining the study’s identified ecological restoration areas with the city’s overall planning, recommendations are made for optimization, including implementing forest ecological protection projects, enhancing the soil and water conservation capacity of the Daheishan Mountain Range, protecting the forest and grass structure, and preventing soil erosion. The salinization extension of agricultural land in the west side region should be intensified, and the development of a wind and sand resistant ecological forest belt should be increased. In order to prevent human activities from destroying the ecological space of agricultural land, it is necessary to expedite the construction of ecological projects such as smart agriculture, while strictly preventing and controlling agricultural practices that destroy habitats, such as straw burning, that persist in certain regions. For ecosystems within cities and towns, urban expansion should be limited, ecological restoration measures, such as the construction of green centers and greenways, should be accelerated for each district and county [58], ecological restoration areas should be protected, and the construction of a protection system with nature reserves as the main body should be accelerated by relying on Jilin Polo Lake National Nature Reserve, Changchun Beihu National Wetland Park, and Changchun Beihu National Nature Reserve. Achieving the aforementioned recommendations will facilitate the transitioning from conventional heavy industries to emerging industries with less reliance on natural resources.

### 4.3. Research Strengths and Limitations

This study deviates from the conventional “passive restoration” in that it emphasizes “active adaptation” and may scientifically identify crucial regions to be conserved and restored by taking into account the ecosystem’s integrity. There are numerous benefits to this study: Initially, the importance of ecosystem services is chosen as the basis for picking ecological sources, and the weights chosen using principal component analysis can account for tradeoffs and synergies across the four ecosystem service functions. To account for the impact of human interference on ecological security patterns, natural variables and human interference factors are chosen next. In addition, graded ecological protection and priority areas are established, as well as specific and targeted optimization measures, which serve as a reference for urban construction and ecological management in Changchun.

The scope of the study is significant for enhancing ecological environment quality, reducing urban sprawl, and attaining sustainable regional development. However, this study has certain defects. Firstly, limited in the artificial border and scope of the present study, it is difficult to reflect the integrity of the ecological security pattern of the national land area in this study. Therefore, in the future, in terms of regions, it is necessary to build an ecological security pattern across cities and strengthen the overall land space restoration; In terms of scale, the ecological restoration area needs to be identified and studied at multiple scales [59]. Second, this study accessing the importance of ecosystem service in Changchun city by selecting four indicators with a one-year cycle, which is not conducive to accessing the stability and variability of different functions offered by different time evolution cycles. The study was constructed from the perspective of ecosystem service supply, so the coupling of supply and human ecological demand should be further considered [60]. Additional research on ecosystem services should also be conducted for the scientific identification of ecological sources. In addition, field exploration and verification of ecological pinch points and barrier points should be addressed in future studies. In doing so, the validity of future study findings will see improvements regarding typical real-world scenarios, and further adapting the study model to site conditions will facilitate more accurate restoration efforts and better protection of sensitive ecological systems.

## 5. Conclusions

This study is based on the construction of an ecological security pattern for home-land security, incorporating the concept of ecological security pattern optimization to identify ecological restoration and graded protection areas, while taking ecological wholeness and ecological function significance into account. The study selects sources by evaluating the importance of ecosystem service functions; fully considers the disturbance of natural systems by human activities; integrates natural environmental factors and human interference factors to construct resistance surfaces; applies circuit theory to extract ecological corridors, ecological pinch points, and barrier points; and maps ecological restoration areas and graded protection areas, with the following major conclusions:

(1) The importance of spatial heterogeneity in ecosystem services including biodiversity conservation, water production, soil conservation, and carbon fixation in Changchun is obvious. There are 4698.92 km^2^ of extremely important areas, accounting for 18.99% of the total city area. These areas are primarily located in the city’s eastern region and are dominated by cultivated land and forests. Among these, Yushu City, the eastern portion of Jiutai District and Erdao District, and the northern portion of Shuangyang District are relatively heavily populated. Through ecological service evaluation and area rationalization allocation, the study identifies 20 ecological source regions with a total area of 3659.28 km^2^, representing 14.79% of ecological land.
(2) The natural resistance surface is rectified using nighttime light intensity data to create an all-encompassing resistance surface. The results indicate that the principal urban region has higher resistance, indicating a propensity for dispersal and that the settlement centers of each county also have higher resistance. Linkage Mapper extracted the lowest cost paths to build ecological corridors, establishing 41 major ecological corridors in the city to form a somewhat dense mesh structure in the south. The centrality of ecological sources was determined, showing that the biggest concentration of ecological sources accumulated in the second channel area and required significant protection. Moreover, 15 ecological barriers and 31 ecological pinch points were identified to guide ecological security pattern conservation and restoration.
(3) The ecological restoration areas were identified, in which prioritized restoration zones consisted of ecological corridor barriers, mostly in the main urban area of Changchun and the urban fringe, which needed to be restored to ensure the flow of materials and energy transport. Prioritized protection zones are located in a small section of the ecological corridor, including Yushu City and Gongchuling City, where urban development must be minimized. Key conservation zones are the core areas of ecological sources with high-centeredness and ecological radiation benefits to the surrounding areas, primarily in the eastern portion of the city. These key areas must be conserved to preserve the circulation of ecological corridors. The general conservation zone contains critical corridors with width information and requires appropriate protection measures to preserve the entire ecological security pattern and enhance overall performance of the ecosystem service.

## Figures and Tables

**Table 1:** Graded assignment of resistance factors and weight assignment.

Resistance Factor	Weights (%)	Tiered Metrics	Resistance Value
land use type	0.38	Forests	10
		Grassland	30
		Cultivated land	100
		Water bodies	200
		Unused land	700
		Construction land	1000
elevation	0.22	0–150	10
		150–275	100
		275–493	300
		493–690	500
		690–965	800
		>965	1000
slope	0.21	0°–7°	10
		7°–14°	100
		14°–23°	200
		23°–34°	600
		>40°	1000
NDVI	0.19	0.82–0.99	10
		0.69–0.82	100
		0.49–0.82	300
		0.21–0.49	1000
		<0.21	1600

**Table 2:** Area and proportion of importance of each function of ecosystem services.

	Biodiversity		Water Production		Soil Conservation		Carbon Fixation	
	Area (km^2^)	Proportion (%)	Area (km^2^)	Proportion (%)	Area (km^2^)	Proportion (%)	Area (km^2^)	Proportion (%)
Less important	2950.87	11.93	724.21	2.93	14,287.97	57.74	2806.37	11.34
Generally important	2359.68	9.54	11,019.89	44.53	5514.05	22.28	419.18	1.69
Slightly important	17,039.78	68.86	4370.64	17.66	3690.71	14.92	2703.69	10.93
Highly important	603.35	2.44	6605.84	26.70	967.55	3.91	17,150.88	69.31
Extremely important	1791.08	7.24	2024.18	8.18	284.48	1.15	1664.65	6.73

**Table 3:** Distribution of the area of each county in the ecological restoration zone.

	Prioritized Restoration Zones (km^2^)	Prioritized Protection Zones (km^2^)	Key Conservation Zones (km^2^)	General Conservation Zones (km^2^)
Nanguan	23.34	8.25	130.28	11.65
Kuancheng	30.79	12.44	44.86	126.08
Chaoyang	1.66	3.07	21.75	10.80
Erdao	20.40	7.82	169.29	49.73
Lvyuan	25.20	4.87	83.59	7.54
Shuangyang	3.11	9.03	33.43	105.73
Jiutai	5.00	0.83	805.21	130.65
Nongan	0.00	12.47	8.68	640.16
Yushu	5.92	0.00	463.49	90.51
Dehui	8.35	11.25	34.33	392.76
Gongzhuling	7.83	39.03	0.02	495.49
total	131.60	109.07	1794.94	2061.11

## Data Availability

Not applicable.

## References

[1] Di Giulio M., Holderegger R., Tobias S. (2009). Effects of Habitat and Landscape Fragmentation on Humans and Biodiversity in Densely Populated Landscapes. J. Environ. Manag..

[2] Solow A.R. (2017). On Detecting Ecological Impacts of Extreme Climate Events and Why It Matters. Philos. Trans. R. Soc. B Biol. Sci..

[3] Tutak M., Brodny J., Bindzár P. (2021). Assessing the Level of Energy and Climate Sustainability in the European Union Countries in the Context of the European Green Deal Strategy and Agenda 2030. Energies.

[4] Zhang J., Cao Y., Ding F., Wu J., Chang I.-S. (2022). Regional Ecological Security Pattern Construction Based on Ecological Barriers: A Case Study of the Bohai Bay Terrestrial Ecosystem. Sustainability.

[5] Dai L., Liu Y., Luo X. (2021). Integrating the MCR and DOI Models to Construct an Ecological Security Network for the Urban Agglomeration around Poyang Lake, China. Sci. Total Environ..

[6] Gonzalez A., Rayfield B., Lindo Z. (2011). The Disentangled Bank: How Loss of Habitat Fragments and Disassembles Ecological Networks. Am. J. Bot..

[7] Rao Y., Dai J., Dai D., He Q., Wang H. (2021). Effect of Compactness of Urban Growth on Regional Landscape Ecological Security. Land.

[8] Zhang Y., Li S., Yan J. (2021). Spatiotemporal Variation of Urban Ecological Security and Its Influencing Factors in North and South of Qinling-Huaihe Region, China. Resour. Environ. Yangtze Basin.

[9] Zhao Y., Kasimu A., Liang H., Reheman R. (2022). Construction and Restoration of Landscape Ecological Network in Urumqi City Based on Landscape Ecological Risk Assessment. Sustainability.

[10] Li J., Xu J., Chu J. (2019). The Construction of a Regional Ecological Security Pattern Based on Circuit Theory. Sustainability.

[11] Gao J., Du F., Zuo L., Jiang Y. (2021). Integrating Ecosystem Services and Rocky Desertification into Identification of Karst Ecological Security Pattern. Landsc. Ecol..

[12] Zhang H., Li J., Tian P., Pu R., Cao L. (2022). Construction of Ecological Security Patterns and Ecological Restoration Zones in the City of Ningbo, China. J. Geogr. Sci..

[13] Yuan Y., Bai Z., Zhang J., Xu C. (2022). Increasing Urban Ecological Resilience Based on Ecological Security Pattern: A Case Study in a Resource-Based City. Ecol. Eng..

[14] Wei Q., Halike A., Yao K., Chen L., Balati M. (2022). Construction and Optimization of Ecological Security Pattern in Ebinur Lake Basin Based on MSPA-MCR Models. Ecol. Indic..

[15] Wang Y., Pan J. (2019). Building Ecological Security Patterns Based on Ecosystem Services Value Reconstruction in an Arid Inland Basin: A Case Study in Ganzhou District, NW China. J. Clean. Prod..

[16] Zhao S., Ma Y., Wang J., You X. (2019). Landscape Pattern Analysis and Ecological Network Planning of Tianjin City. Urban For. Urban Green..

[17] Peng J., Yang Y., Liu Y., Hu Y., Du Y., Meersmans J., Qiu S. (2018). Linking Ecosystem Services and Circuit Theory to Identify Ecological Security Patterns. Sci. Total Environ..

[18] Samways M.J., Bazelet C.S., Pryke J.S. (2010). Provision of Ecosystem Services by Large Scale Corridors and Ecological Networks. Biodivers. Conserv..

[19] Liquete C., Kleeschulte S., Dige G., Maes J., Grizzetti B., Olah B., Zulian G. (2015). Mapping Green Infrastructure Based on Ecosystem Services and Ecological Networks: A Pan-European Case Study. Environ. Sci. Policy.

[20] Lookingbill T.R., Gardner R.H., Ferrari J.R., Keller C.E. (2010). Combining a Dispersal Model with Network Theory to Assess Habitat Connectivity. Ecol. Appl..

[21] Peng W., Zhou J. (2019). Development of Land Resources in Transitional Zones Based on Ecological Security Pattern: A Case Study in China. Nat. Resour. Res..

[22] Wang D., Chen J., Zhang L., Sun Z., Wang X., Zhang X., Zhang W. (2019). Establishing an Ecological Security Pattern for Urban Agglomeration, Taking Ecosystem Services and Human Interference Factors into Consideration. PeerJ.

[23] Jin X., Wei L., Wang Y., Lu Y. (2021). Construction of Ecological Security Pattern Based on the Importance of Ecosystem Service Functions and Ecological Sensitivity Assessment: A Case Study in Fengxian County of Jiangsu Province, China. Environ. Dev. Sustain..

[24] Yu Q., Yue D., Wang Y., Kai S., Fang M., Ma H., Zhang Q., Huang Y. (2018). Optimization of Ecological Node Layout and Stability Analysis of Ecological Network in Desert Oasis: A Typical Case Study of Ecological Fragile Zone Located at Deng Kou County (Inner Mongolia). Ecol. Indic..

[25] Huang L., Wang J., Cheng H. (2022). Spatiotemporal Changes in Ecological Network Resilience in the Shandong Peninsula Urban Agglomeration. J. Clean. Prod..

[26] Li Q., Zhou Y., Yi S. (2022). An Integrated Approach to Constructing Ecological Security Patterns and Identifying Ecological Restoration and Protection Areas: A Case Study of Jingmen, China. Ecol. Indic..

[27] Su X., Zhou Y., Li Q. (2021). Designing Ecological Security Patterns Based on the Framework of Ecological Quality and Ecological Sensitivity: A Case Study of Jianghan Plain, China. Int. J. Environ. Res. Public Health.

[28] Yu C., Liu D., Feng R., Tang Q., Guo C. (2021). Construction of Ecological Security Pattern in Northeast China Based on MCR Model. Acta Ecol. Sin..

[29] Wang X., Ding Y., Wang S. (2018). Evaluation of Ecological Suitability of Urban Construction Land in Changchun City Based on ANP-GIS. Res. Soil Water Conserv..

[30] Nematollahi S., Fakheran S., Kienast F., Jafari A. (2020). Application of InVEST Habitat Quality Module in Spatially Vulnerability Assessment of Natural Habitats (Case Study: Chaharmahal and Bakhtiari Province, Iran). Environ. Monit. Assess.

[31] Berta Aneseyee A., Noszczyk T., Soromessa T., Elias E. (2020). The InVEST Habitat Quality Model Associated with Land Use/Cover Changes: A Qualitative Case Study of the Winike Watershed in the Omo-Gibe Basin, Southwest Ethiopia. Remote Sens..

[32] Hao Y., Zhang N., DU Y.J., Wang Y.H., Zheng Y.D., Zhang C.C. (2019). Construction of Ecological Security Pattern Based on Habitat Quality in Tang County, Hebei, China. J. Appl. Ecol..

[33] Wang Y., Ji Y., Yu H., Lai X. (2022). Measuring the Relationship between Physical Geographic Features and the Constraints on Ecosystem Services from Urbanization Development. Sustainability.

[34] Okou F.A.Y., Tente B., Bachmann Y., Sinsin B. (2016). Regional Erosion Risk Mapping for Decision Support: A Case Study from West Africa. Land Use Policy.

[35] Renard K.G., Freimund J.R. (1994). Using Monthly Precipitation Data to Estimate the R-Factor in the Revised USLE. J. Hydrol..

[36] Liu X., Li X., Liang X., Shi H., Ou J. (2019). Simulating the Change of Terrestrial Carbon Storage in China Based on the FLUS-InVEST Model. Trop. Geogr..

[37] Lin Q., Mao J., Wu J., Li W., Yang J. (2016). Ecological Security Pattern Analysis Based on InVEST and Least-Cost Path Model: A Case Study of Dongguan Water Village. Sustainability.

[38] Loc H.H., Park E., Thu T.N., Diep N.T.H., Can N.T. (2021). An Enhanced Analytical Framework of Participatory GIS for Ecosystem Services Assessment Applied to a Ramsar Wetland Site in the Vietnam Mekong Delta. Ecosyst. Serv..

[39] Gao S., Sun H., Cao G., Zhao L., Wang R., Xu M. (2018). Dynamic Assessment of Island Ecological Security under Urbanization: A Case Study of Pingtan Island in the Southeast Coast of China. Environ. Earth Sci..

[40] Zhang C., Jia C., Gao H., Shen S. (2022). Ecological Security Pattern Construction in Hilly Areas Based on SPCA and MCR: A Case Study of Nanchong City, China. Sustainability.

[41] Dong R., Zhang X., Li H. (2019). Constructing the Ecological Security Pattern for Sponge City: A Case Study in Zhengzhou, China. Water.

[42] Li Y., Liu W., Feng Q., Zhu M., Yang L., Zhang J., Yin X. (2023). The Role of Land Use Change in Affecting Ecosystem Services and the Ecological Security Pattern of the Hexi Regions, Northwest China. Sci. Total Environ..

[43] Zhao X., Yue Q., Pei J., Pu J., Huang P., Wang Q. (2021). Ecological Security Pattern Construction in Karst Area Based on Ant Algorithm. Int. J. Environ. Res. Public Health.

[44] Guo R., Wu T., Liu M., Huang M., Stendardo L., Zhang Y. (2019). The Construction and Optimization of Ecological Security Pattern in the Harbin-Changchun Urban Agglomeration, China. Int. J. Environ. Res. Public Health.

[45] Pickett S.T.A., Cadenasso M.L., Rosi-Marshall E.J., Belt K.T., Groffman P.M., Grove J.M., Irwin E.G., Kaushal S.S., LaDeau S.L., Nilon C.H. (2017). Dynamic Heterogeneity: A Framework to Promote Ecological Integration and Hypothesis Generation in Urban Systems. Urban Ecosyst..

[46] Chen Y., Huang L. (2018). A Scaling Approach to Evaluating the Distance Exponent of the Urban Gravity Model. Chaos Solitons Fractals.

[47] Li S., Xiao W., Zhao Y., Lv X. (2020). Incorporating Ecological Risk Index in the Multi-Process MCRE Model to Optimize the Ecological Security Pattern in a Semi-Arid Area with Intensive Coal Mining: A Case Study in Northern China. J. Clean. Prod..

[48] Huang J., Hu Y., Zheng F. (2020). Research on Recognition and Protection of Ecological Security Patterns Based on Circuit Theory: A Case Study of Jinan City. Environ. Sci. Pollut. Res..

[49] Wang J., Liu C., Zhang S. (2022). Ecological Security Pattern of Typical Counties in Northern Sand Prevention Belts. Acta Ecol. Sin..

[50] Chen X., Lou J., Wang Y. (2020). Evolution and Dynamic Simulation of the Temporal-Spatial Pattern of Urban Resilience in Harbin-Changchun Urban Group. Sci. Geogr. Sin..

[51] Guo X., Chang Q., Liu X., Bao H., Zhang Y., Tu X., Zhu C., Lv C., Zhang Y. (2018). Multi-Dimensional Eco-Land Classification and Management for Implementing the Ecological Redline Policy in China. Land Use Policy.

[52] Liu Z., Huang Q., Tang G. (2021). Identification of Urban Flight Corridors for Migratory Birds in the Coastal Regions of Shenzhen City Based on Three-Dimensional Landscapes. Landsc. Ecol..

[53] Fu Y., Shi X., He J., Yuan Y., Qu L. (2020). Identification and Optimization Strategy of County Ecological Security Pattern: A Case Study in the Loess Plateau, China. Ecol. Indic..

[54] Liu W., Xu H., Zhang X., Jiang W. (2022). Green Infrastructure Network Identification at a Regional Scale: The Case of Nanjing Metropolitan Area, China. Forests.

[55] Ran Y., Lei D., Li J., Gao L., Mo J., Liu X. (2022). Identification of Crucial Areas of Territorial Ecological Restoration Based on Ecological Security Pattern: A Case Study of the Central Yunnan Urban Agglomeration, China. Ecol. Indic..

[56] Chen X., Li F., Li X., Liu H., Hu Y., Hu P. (2021). Integrating Ecological Assessments to Target Priority Restoration Areas: A Case Study in the Pearl River Delta Urban Agglomeration, China. Remote Sens..

[57] Liu Y., Liu S., Wang F., Liu H., Li M., Sun Y., Wang Q., Yu L. (2022). Identification of Key Priority Areas under Different Ecological Restoration Scenarios on the Qinghai-Tibet Plateau. J. Environ. Manag..

[58] Jeong D., Kim M., Song K., Lee J. (2021). Planning a Green Infrastructure Network to Integrate Potential Evacuation Routes and the Urban Green Space in a Coastal City: The Case Study of Haeundae District, Busan, South Korea. Sci. Total Environ..

[59] Dong J., Peng J., Xu Z., Liu Y., Wang X., Li B. (2021). Integrating Regional and Interregional Approaches to Identify Ecological Security Patterns. Landsc. Ecol..

[60] Zhang Y., Zhao Z., Fu B., Ma R., Yang Y., LU Y., Wu X. (2022). Identifying Ecological Security Patterns Based on the Supply, Demand and Sensitivity of Ecosystem Service: A Case Study in the Yellow River Basin, China. J. Environ. Manag..

